# Correction: Affective bias as a rational response to the statistics of rewards and punishments

**DOI:** 10.7554/eLife.32902

**Published:** 2017-10-19

**Authors:** Erdem Pulcu, Michael Browning

Pulcu E, Browning M. 2017. Affective bias as a rational response to the statistics of rewards and punishments. *eLife* **6**:e27879. doi: 10.7554/eLife.27879.

Published 04, October 2017

In the published article there is an error in the Figure key for Figure 3—the lines which are labelled as “win outcome, wins stable” and “win outcome, wins volatile” are the wrong way round. These lines are described (correctly) in the legend of the article.

The Corrected Figure 3 is shown here:

**Figure 1:**
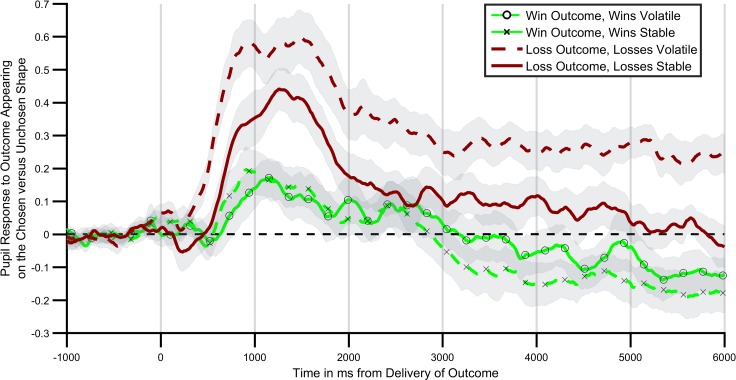


The originally published Figure 3 is also shown for reference:

**Figure 2:**
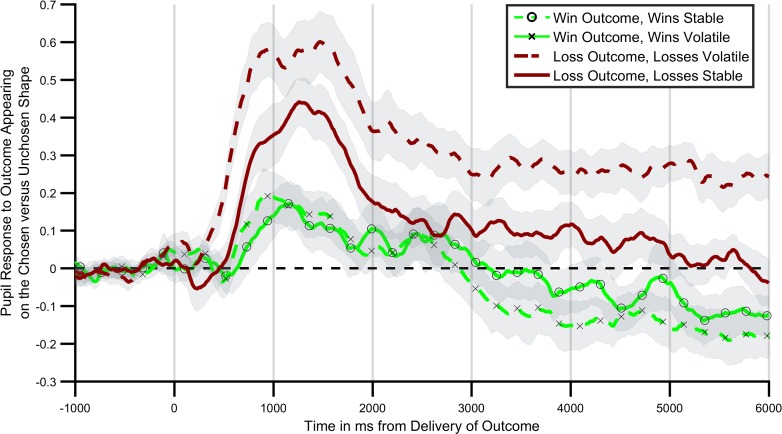


The article has been corrected accordingly.

